# Bayesian inference informed by parameter subset selection for a minimal PBPK brain model

**DOI:** 10.1098/rsta.2024.0219

**Published:** 2025-03-13

**Authors:** Kamala Dadashova, Ralph C. Smith, Mansoor A. Haider, Brian J. Reich

**Affiliations:** ^1^ Department of Mathematics, North Carolina State University, Raleigh, NC 27695, USA; ^2^ Department of Statistics, North Carolina State University, Raleigh, NC 27695, USA

**Keywords:** parameter subset selection, Bayesian inference, physiologically based pharmacokinetic modelling, parameter identifiability

## Abstract

Physiologically based pharmacokinetic (PBPK) models use a mechanistic approach to delineate the processes of the absorption, distribution, metabolism and excretion of biological substances in various species. These models generally comprise coupled systems of ordinary differential equations involving multiple states and a moderate to a large number of parameters. Such models contain compartments corresponding to various organs or tissues in the body. Before employing the models for treatment, the quantification of uncertainties for the parameters, based on *a priori* information or data for a specific response, is necessary. This requires the determination of identifiable parameters, which are uniquely determined by data, and uncertainty analysis based on frequentist or Bayesian inference. We introduce a strategy to integrate parameter subset selection, based on identifiability analysis, with Bayesian inference. This approach further refines the subset of identifiable parameters, quantifies parameter and response uncertainties, enhances model prediction and reduces computational cost.

This article is part of the theme issue ‘Uncertainty quantification for healthcare and biological systems (Part 1)’.

## 1. Introduction

Physiologically based pharmacokinetic (PBPK) models combine physiological and biochemical information to describe the pharmacokinetics (PK) of an anatomical process of interest [1]. Such models provide a mechanistic framework to understand how drugs and other chemical components move through the body [2].

Since these models are based on compartmental structure, they can facilitate simulations and predictions for drug development by partitioning complex biological systems into their associated components. They usually include numerous parameters and model responses to describe the compartments or sub-compartments in the model. The parameters of PBPK models can be classified into various categories, each having an important role in predicting the PK of chemical substances [3]. There are anatomical (e.g. organ volumes and tissue masses), physiological (e.g. tissue volumes and blood flow rates), chemical-specific (e.g. partition coefficients and diffusivity) and drug-specific (e.g. clearance rate and tissue to plasma partition coefficients) parameters [4].

Determination of these parameters and quantification of uncertainties is challenging in PBPK models for several reasons. These include inherent uncertainties owing to variability in physiological or biological systems, measurement errors and limited available data. Hence, many parameters cannot be directly measured from the experimental data. For this reason, it is important to understand how variation in parameters affects the model responses across the time domain of interest, i.e. the local sensitivities of individual responses to each parameter in the model. Local identifiability analysis is an important approach, based on local sensitivities, which can be employed to partition parameters into identifiable and non-identifiable parameter subsets. These subsets might differ for different time regimes, variables of interest or nominal parameter values. There are various local sensitivity-based techniques available in the literature to determine identifiable parameters. Whereas some use data to compute sensitivity derivatives of the residuals [5–7], others can be used in the absence of data [8]. PBPK models often involve parameters that can exhibit varying identifiability depending on the specific time points and model variables of interest. Local identifiability analysis is particularly appropriate for capturing these variations, as it evaluates parameter sensitivity around nominal values, which can shift in different time regimes or under different model responses.

Robust and accurate identifiability analysis for PBPK model parameters is a key step before conducting statistical inference for parameter estimation or uncertainty quantification. Whereas a traditional frequentist approach is practical for parameter estimation or construction of confidence intervals, it has limitations in incorporating prior knowledge about the parameters. Bayesian inference offers several advantages for parameter estimation in PBPK models compared to a frequentist approach. The benefits include [9,10] (i) the integration of prior knowledge about parameters, which can be particularly helpful for sparse or noisy data; (ii) the construction of posterior distribution, which provides the integration of prior knowledge or expert opinion about parameter values with observed data and often leads to more accurate and reliable parameter estimates in comparison to using data alone; and (iii) the ability to directly propagate parameter distributions through models to construct prediction intervals for responses or quantities of interest.

In this paper, we introduce a method for integrating parameter subset selection (PSS), which quantifies parameter identifiability, with Bayesian inference in the context of a minimal PBPK (mPBPK) model of the brain for antibody therapeutics [11]. The inclusion of non-identifiable parameters in Bayesian analysis can yield poor convergence and mixing of parameter chains, a high posterior variance and biased estimates in the absence of a highly informative and reliable prior distribution. Furthermore, algorithms for computing the posterior distribution of parameters become computationally expensive when the number of parameters is large. One solution is to fix non-identifiable parameters at their nominal values [10], which are generally taken to be a standard or reference value for a particular parameter or variable. We suggest using a local identifiability-based PSS as an initial screening to determine subsets of identifiable and non-identifiable parameters. This step simplifies the complex models by reducing the dimension of parameter space. However, it is local in nature and does not quantify parameter uncertainties.

Bayesian inference provides a probabilistic framework that inherently incorporates statistical parameter interactions and uncertainties. It can reveal complex dependencies and interactions between parameters by providing a joint posterior distribution of the parameters. We also employ dimensional analysis for the subset of parameter candidates to investigate potential algebraic relations among them. Whereas PSS helps reduce complexity before employing Bayesian inference, the combination of Bayesian inference and dimensional analysis further refines the subset of identifiable parameters and quantifies uncertainties. This iterative process utilizes the strengths of these three approaches, leading to a more accurate identifiable parameter subset, inference of parameter distributions and response predictions with quantified uncertainties.

We summarize PSS techniques, aspects of dimensional analysis relevant to our study, and some foundations of Bayesian inference in §2. The integration of PSS into Bayesian inference and an algorithm for this iterative process is described in §3. Section 4 provides information about synthetic data, dimensional analysis for the mPBPK model and a prior distribution for determined parameters. In §5, we provide our results, which are presented for a key response of interest in the model (concentration in plasma). We end with a brief discussion and conclusions in §6.

## 2. Background

The mPBPK model can be generally expressed as


(2.1) $$\begin{array}{l} \frac{dx}{dt}=g(t,x(t),\theta ),\;x(t_{0})=x_{0}(\theta ). \end{array}$$

**Figure 1.**
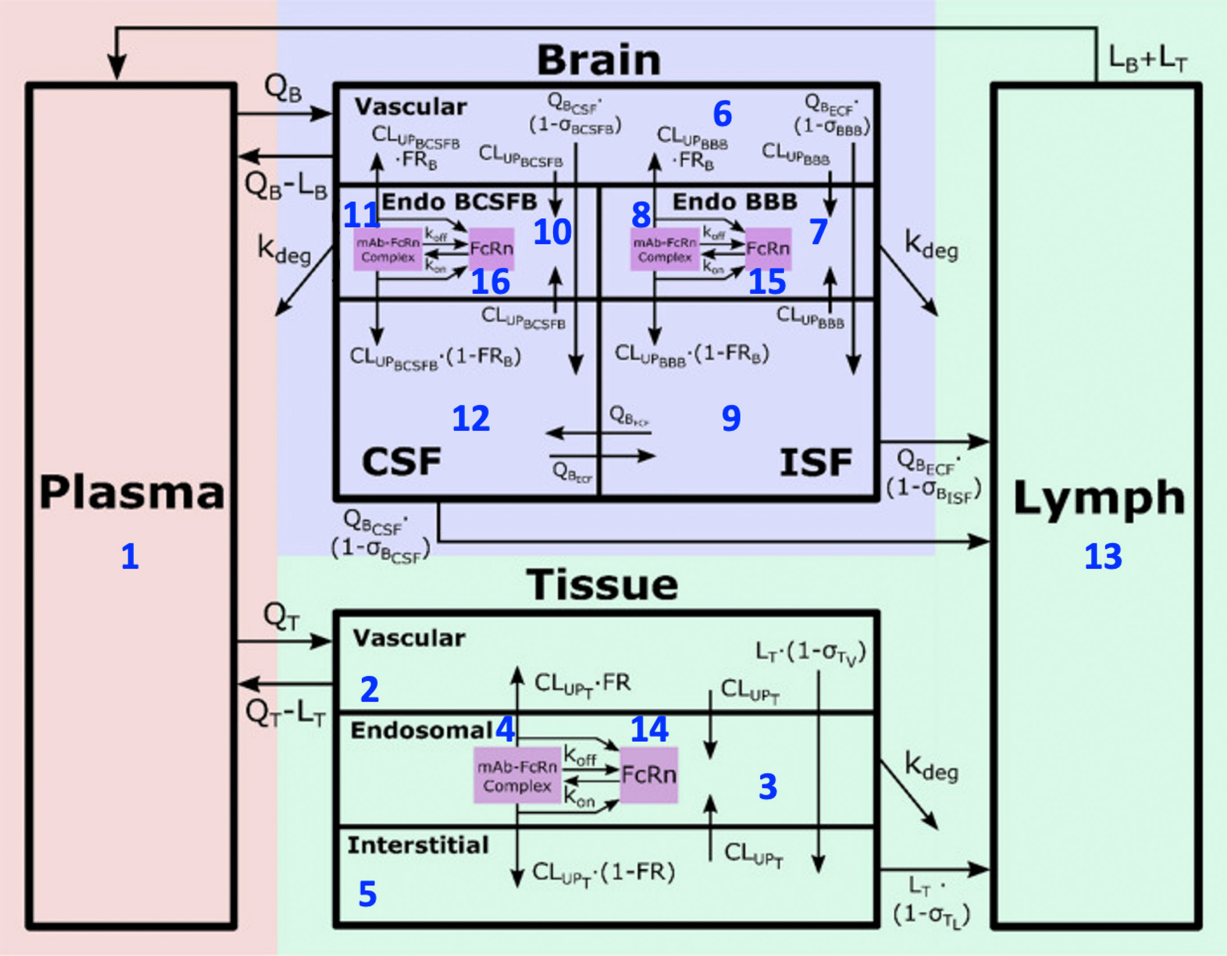
Brain mPBPK model structure [11]. Compartments included in the model represent antibody concentrations in (1) plasma, (2) tissue vascular, (3) tissue endosome (unbound), (4) tissue endosome (FcRnbound), (5) tissue interstitium, (6) brain vascular, (7) blood–brain barrier (BBB) endosome (unbound), (8) BBB endosome (FcRnbound), (9) brain interstitium, (10) blood-cerebrospinal fluid barrier (BCSFB) endosome (unbound), (11) BCSFB endosome (FcRnbound), (12) brain CSF, (13) lymph, (14) FcRn concentration in tissue endosome, (15) FcRn BBB endosome and (16) FcRn BCSFB endosome. (Permission to be obtained prior to publication.)

Here, $𝒙(t)=[x_{1},\ldots ,x_{m}{]}^{T}$ are the model states or responses, $𝜽=[\theta_{1},\ldots ,\theta_{p}{]}^{T}$ contains the full set of p model parameters and g is the right-hand side of a system of ordinary differential equations that describes the model dynamics by defining the rates of change of the state variables in the system [11]. In our mPBPK analysis in §2a, there are m=16 states and p=31 parameters in the model. The compartments, sub-compartments and parameters in the mPBPK model are provided in figure 1.

### (a) PSS

We employ the parameter identifiability results for a mPBPK brain model based on the PSS algorithms published in [8]. The PSS algorithm starts with a local sensitivity analysis, which quantifies how changes in each parameter affect model responses. Local sensitivities are evaluated at n discrete time points using the partial derivatives evaluated at parameter nominal values $\theta^{*}=(\theta_{1}^{*},\cdots ,\theta_{p}^{*}$),


(2.2) $$\begin{array}{r} s_{j}(t_{i})={\frac{\partial x(t_{i})}{\partial \hat{\theta_{j}}}|}_{\theta =\theta^{*}},\;i=1,\ldots ,n,\;j=1,\ldots ,p, \end{array}$$

of the model responses $x(t_{i})$ with respect to each parameter. Here, $s_{j}$ is the local sensitivity with respect to the jth parameter, where we use a log transformation ($\hat{\theta_{j}}=\log\;\theta_{j}$), $t_{i}$ is the ith time point and n is the number of time steps (n=1001) in our example. Note that nominal parameter values are physiologically accepted values obtained either through optimization using data or previous results reported in the literature. We employed the nominal values reported in [11]. The sensitivities of model responses depend on the specific response of interest, the times at which the response is calculated and the nominal values themselves.

A sensitivity matrix for the considered concentration is constructed using these local sensitivities. By applying a singular value decomposition to the n×p sensitivity matrix, the algorithm specifies parameters having the most important effect on model outputs, ranking them based on their associated singular values. If the square of the ratio of the largest to the smallest singular value is greater than a prescribed threshold value η, all parameters are deemed to be identifiable. Otherwise, the position of the component with the largest magnitude in the singular vector is determined, which corresponds to the least identifiable parameter. We note that PSS is a linear algebra technique that is used to sequentially eliminate the least identifiable parameter and determine a subset of parameters that are identifiable [8,12]. The associated sequence of subsets of parameter candidates is denoted by $A=\{A_{1},\ldots ,A_{k}\}$ with each corresponding to the threshold values of $\eta =\{\eta_{1},\ldots ,\eta_{k}\}$. Whereas there is no precise strategy to choose threshold values η in the PSS algorithm, here we choose candidate values so that each larger subset contains one additional parameter, i.e. $A_{i}\subset A_{i+1}$ and $|A_{i+1}|=|A_{i}|+1$, i=1,…,k−1. We then employ these candidates in a Bayesian refinement algorithm to construct a set of parameters suitable for subsequent uncertainty quantification.

### (b) Dimensional analysis

Dimensional analysis is a classical, algebraic technique in mathematical modelling that enforces inherent constraints among dimensions (or units) in mathematical relations; see §3a. It is commonly applied to a general relation representing a model or system response in terms of its parameters and/or independent variables. Dimensional analysis reduces the number of independent mathematical variables on which the response depends relative to the total number of parameters or variables in the general relation. This is accomplished via a combination of variables matching the units of the response multiplied by an unknown function of a set of ‘dimensionless products’. Each such dimensionless product is a unitless combination of model parameters or independent variables. Here, we will apply basic principles of dimensional analysis to illustrate how, in the process of combining PSS and Bayesian inference, some structural parameter dependencies can be determined, among the subset of identifiable parameters.

### (c) Bayesian inference

Bayesian inference quantifies parametric uncertainty by treating the unknown parameters θ as random variables. In the Bayesian framework, the prior distribution of the parameters is combined with the likelihood of the observed data to construct a posterior distribution for parameters. The prior distribution $\pi_{0}(\theta )$ represents beliefs regarding the parameters in the model before observing the data. The choice of the prior distribution is subjective [13]. The likelihood function p(y|θ) links the observed data to unknown parameters, where 𝒚 denotes a realization of a random observation vector Y. The posterior distribution is the updated probability of a parameter after observing data, incorporating prior beliefs and the likelihood of the observed data. It is used to make a prediction and decision based on both prior knowledge and new evidence. Bayes’ rule is used to link the posterior distribution with the prior distribution and the likelihood function [10]


(2.3) $$\begin{array}{r} \pi (\theta |y)=\frac{p(y|\theta )\pi_{0}(\theta )}{\pi_{Y}(y)}, \end{array}$$

where $\pi_{Y}(y)=\int_{R^{p}}p(y|\theta )\pi_{0}(\theta )d\theta$, and p is the number of parameters.

The likelihood function is determined by the observation model for the data $Y=(Y_{1},\cdots ,Y_{n})$. We employ the additive observation model


(2.4) $$\begin{array}{r} Y_{i}=f(t_{i},\theta )+\epsilon_{i}, \end{array}$$

where $f(t_{i},\theta )$ is the response of interest obtained solving equation (2.1), and $\epsilon_{i}$ is an observation error, drawn from a Gaussian distribution $\epsilon_{i}\overset{\text{iid}}{∼}N(0,\sigma^{2})$. In our example, the time domain of interest is taken to be 0–1000 h, and the total number of observations is n=1001.

Markov chain Monte Carlo (MCMC) techniques are commonly used to estimate the posterior distribution of the parameters. MCMC algorithms produce samples from the posterior distribution that can be used to approximate the posterior. Various MCMC algorithms are available in the literature, such as Hamiltonian Monte Carlo algorithms [14], delayed rejection adaptive metropolis (DRAM) [15], Metropolis and Metropolis–Hastings [16] or DiffeRential Evolution Adaptive Metropolis [17]. We employ DRAM in this investigation. This algorithm improves upon traditional MCMC techniques by incorporating two key features: delayed rejection and adaptive scaling of the proposal distribution. Delayed rejection helps to explore the parameter space more efficiently by allowing for a second, smaller proposal if the first one is rejected, reducing the risk of getting stuck in low-probability regions. The adaptive scaling adjusts the proposal distribution during sampling to optimize acceptance rates, making DRAM more robust to variations in parameter scales. These features make it particularly well suited for complex, high-dimensional models where traditional MCMC might struggle.

After constructing MCMC samples, it is important to verify convergence of the chains. This can be accomplished either by examining trace plots associated with each parameter or using statistical diagnostics such as lag-k auto-correlation [18], Gelman–Rubin statistics [19], Geweke statistics [12] or effective sample size (ESS) [20]. Good chain mixing is important for the complete exploration of parameter space since it prevents the algorithm from stagnating in various regions. If poor mixing or non-convergence are detected, one can employ strategies such as increasing the number of chain iterations M, utilizing a more advanced MCMC algorithm, choosing different initial values for the parameters, selecting more informative priors or simplifying the model [10,13].

A posterior predictive distribution (PPD) is constructed to assess how well a model predicts new data. The PPD can be formulated as


(2.5) (4)π∗(Y∗|y)=∫Rpp(Y∗|θ)π(θ|y) dθ,

where p is the likelihood and π is a posterior distribution [10,13]. To sample $Y^{*}|y∼\pi^{*}(Y^{*}|y)$, one first draws samples from posterior distribution $\tilde{\theta }∼\pi (\theta |y)$, which is followed by a prediction $Y^{*}|\tilde{\theta }$ using likelihood function $p(Y|\tilde{\theta }).$ We repeat this process many times to approximate PPD and construct a Bayesian prediction interval, which quantifies the probability of future computed or experimental observations.

## 3. Integrating PSS with Bayesian inference

We introduce an iterative method in this section that combines PSS and dimensional analysis with Bayesian inference to improve the overall modelling and inference process. The PSS algorithm is applied to initially screen the full set of model parameters to determine a subset of the identifiable parameters that can be estimated from the data. This reduces the number of parameters that need to be estimated in the subsequent Bayesian analysis.

Whereas PSS is an initial screening for obtaining information about identifiable parameters, it is local in nature about employed nominal values and quantifies statistical dependencies. With the proposed approach, we begin with the smallest subset of parameters $A_{1}$ corresponding to the largest threshold value $\eta_{1}$ and employ DRAM to compute the posterior and joint posterior distribution of parameters. To determine whether Bayesian analysis is satisfactory, we evaluate the following criteria:

(C1) **Absence of algebraic relations in joint posterior pairwise plots**: In some cases, PSS can miss algebraic dependencies that are often manifested as single-valued pairwise posterior distributions. When this occurs, we can use dimensional analysis to determine possible algebraic relations among the identifiable parameters. We then fix one of the parameters in the algebraic relation, based on specific features of the model.

(C2) **Informed posterior**: Another important insight provided by Bayesian inference occurs when the marginal posterior distribution of a parameter is approximately the same as its prior distribution. In such cases, the data and model do not inform the prior, and we conclude that this parameter is not identifiable and needs to be fixed at its nominal value.

(C3) **MCMC convergence**: This must be examined via trace plots or statistical metrics, such as ESS, indicating the number of independent samples that provide the same information as the correlated sample. A higher (1000 or above) ESS value indicates better mixing and lower auto-correlation, leading to more reliable statistical inference.

These three criteria are incorporated into an iterative procedure, outlined in Algorithm 1. This algorithm adjusts the current subset of parameters, denoted by $A_{\text{current}}$, by excluding a parameter when it is fixed at its nominal value. The next step involves verifying that the three criteria are met by rerunning the Bayesian inference on the subset with one fewer parameter. We then proceed to the next (smaller) threshold value (η), which corresponds to the next (larger) subset of parameter candidates ($A_{i}\to A_{i+1}$), which has one additional parameter. When this iterative procedure is completed for all subsets $A_{1},\ldots ,A_{k}$, we select the largest refined parameter subset determined for all iterations; these subsets are stored in a list $A_{\text{final}}$.

The outcome of this process leads to a refined set of identifiable parameters having marginal posterior distributions that can be employed in uncertainty quantification.


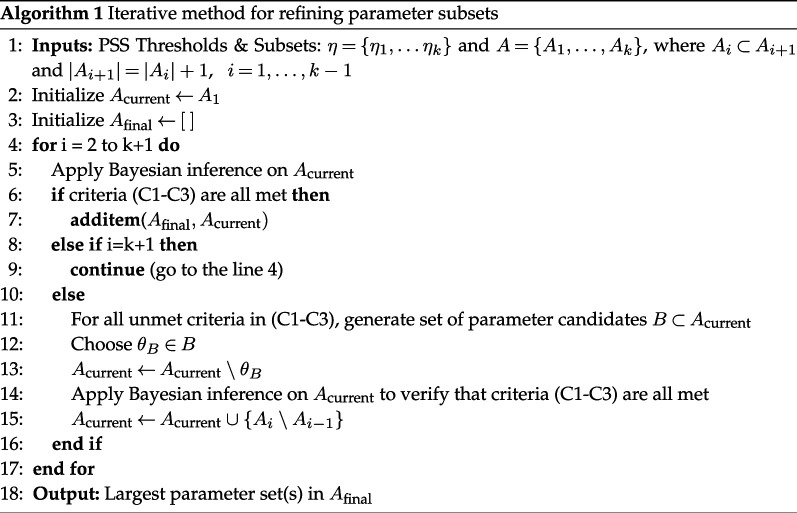


## 4. mPBPK example

To illustrate the iterative approach in §3, we analyse synthetic data for the mPBPK model. The specific target concentration of antibodies varies depending on the biological compartment in which they are measured. These compartments include plasma, brain interstitial fluid and cerebrospinal fluid. This investigation focuses on the concentration levels in the plasma, and we generate synthetic data for this response using equation (2.4). The error variance $\sigma^{2}$ is taken to be 2% of the maximum value in the time domain for plasma concentration $C_{P}$. We have also analysed other noise levels, including 1, 5 and 10%. We select 2% of the maximum value in the response for this example because it explores the variability in the response without establishing too much variation.

**Figure 2.**
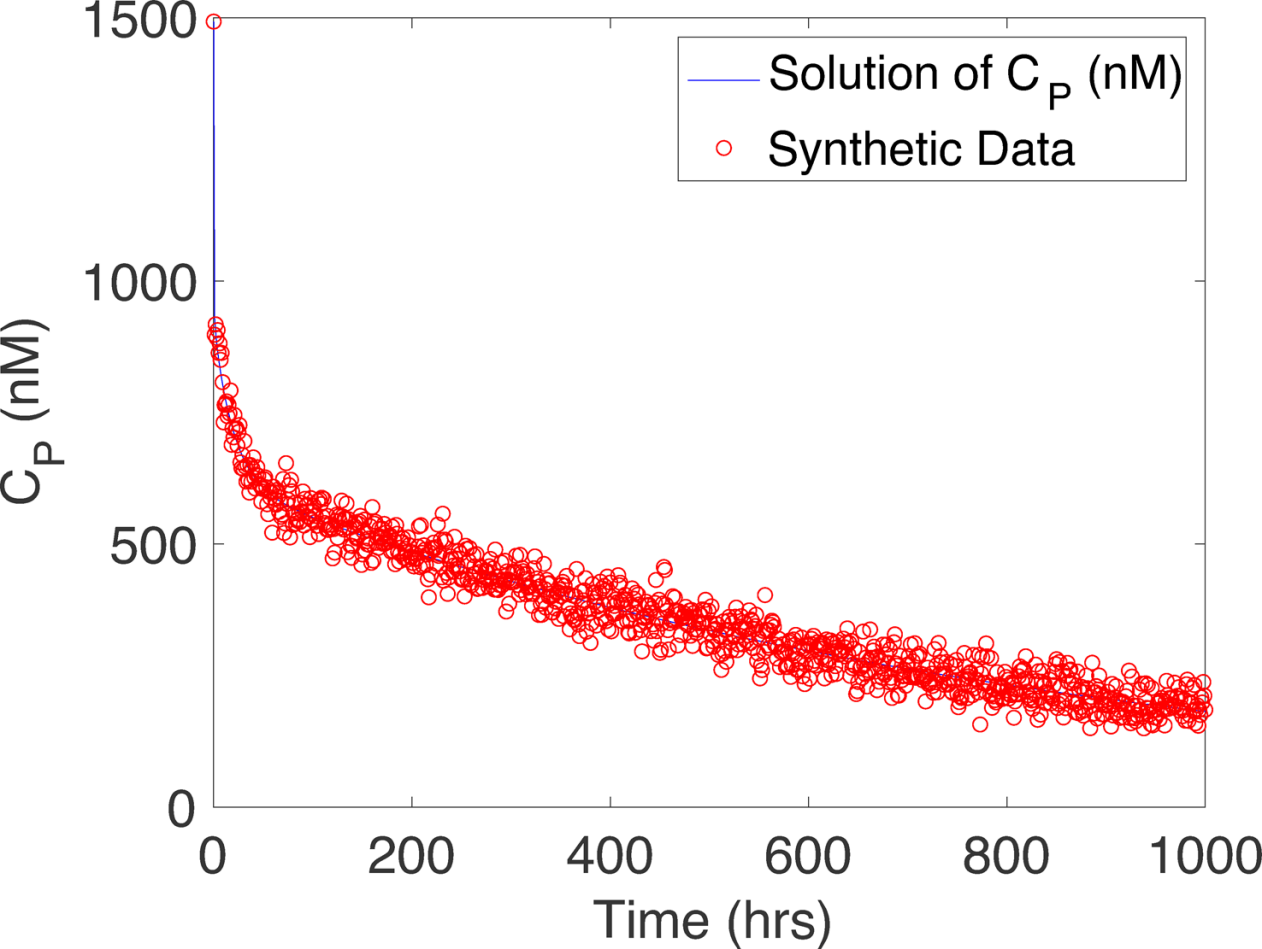
Synthetic data and plasma concentration curve $C_{P}$.

Furthermore, typical magnitudes of $C_{P}$ are of the order of $10^{-9}$ mole (M). The small magnitude of this concentration can pose computational challenges, e.g. the sum of squared errors in DRAM may approach machine epsilon. To avoid numerical instability, we convert the response from molar concentration (M) to nanomolar concentration (nM) and compare the outcomes in both measurement units. We plot the synthetic dataset with the targeted response $C_{P}$ in figure 2.

Before applying our method to the mPBPK model, we conduct a preliminary dimensional analysis and provide prior distributions for the parameters.

### (a) Dimensional analysis

We perform dimensional analysis to determine whether there are algebraic relationships among the identifiable parameters in the mPBPK model. Since the threshold value of $\eta =10^{-12}$ corresponds to the largest parameter subset in [8], we use this specific subset for our dimensional analysis. We have 10 parameter candidates: $V_{P}$, $\sigma_{T_{V}}$, $k_{deg}$, $V_{T_{E}}$, $V_{T_{I}}$, FR, $\sigma_{BCSFB}$, $L_{T}$, $\sigma_{BBB}$, $V_{T_{V}}$. The unit of volume is litres (l), $k_{deg}$ is inverse hours (1/h), $L_{T}$ is litres per hour (l/h), whereas $\sigma_{BBB}$, $\sigma_{BCSFB}$, $\sigma_{T_{V}}$, FR are all unitless. The plasma concentration ($C_{P}$) has units of moles per litre (M/l).

The response consists of the plasma concentration $C_{P}$ yields the relation


(4.1) $$C_{P}=F(C_{0},L_{T},V_{T_{V}},V_{T_{I}},V_{T_{E}},V_{P},k_{deg},FR,\sigma_{BBB},\sigma_{BCSFB},\sigma_{T_{V}}),$$

where $C_{0}$ is the initial plasma concentration value.

Using square brackets to denote the dimensions of each quantity, equation (4.1) can be rewritten as


(4.2) $$\begin{array}{r} {}[C_{P}]=[C_{0}{]}^{a}[L_{T}{]}^{b}[V_{T_{V}}{]}^{c}[V_{T_{I}}{]}^{d}[V_{T_{E}}{]}^{e}[V_{P}{]}^{f}[k_{deg}{]}^{g}G(FR,\sigma_{BBB},\sigma_{BCSFB},\sigma_{T_{V}}), \end{array}$$

where G is an unknown function of the model parameters that are unitless. Based on the units of each parameter and concentration, we obtain the system of linear equations


(4.3) $$\begin{array}{l} \left\{ \begin{array}{l} a=1,\\ -a+b+c+d+e+f=-1,\\ -b-g=0. \end{array}\right.\; \end{array}$$

The dimension of the null space of the coefficient matrix is the number of new dimensionless products, i.e. in addition to the unitless model parameters. A parametric solution of this system is a=1, g=−b, d=−b−c−f−e, where b,c,f,e are free parameters. Substituting this parameterization into equation (4.2) yields the reduced relation


(4.4) $$\begin{array}{r} C_{P}=C_{0}H\left(\frac{L_{T}}{V_{T_{I}}k_{deg}},\frac{V_{T_{V}}}{V_{T_{I}}},\frac{V_{T_{E}}}{V_{T_{I}}},\frac{V_{P}}{V_{T_{I}}},FR,\sigma_{BBB},\sigma_{BCSFB},\sigma_{T_{V}}\right), \end{array}$$

where H is a (new) unknown function that accounts for four new dimensionless products in the relation. Note that alternate parameterizations of the linear system solution yield different sets of four dimensionless products; each set can be obtained as multiplicative combinations of the four dimensionless products in equation (4.4).

As we observe from the first argument of H in equation (4.4), the parameters $L_{T},$$V_{T_{I}}$ and $k_{deg}$ cannot all be estimated at the same time based on data for the plasma concentration $C_{P}$.

### (b) Prior distributions for parameters

Inputs to the DRAM algorithm include the prior distributions for the parameters. Based on the range of the parameters in [11], $\sigma_{T_{V}}$, $\sigma_{BCSFB}$ and $\sigma_{BBB}$ are switch coefficients ranging from 0 to 1, which define the proportion going to each compartment. The remaining parameters must be positive. Since we do not have additional information about the parameters, we choose a uniform prior distribution. The units of the largest subset of parameters, along with their nominal values and prior distributions are listed in table 1.

**Table 1.** Identifiable parameters, their corresponding units, nominal values (${𝜽}^{*}$) [11] and prior distributions.

parameter	units	nominal values	prior distribution
$\sigma_{T_{V}}$	—	0.92	U(0,1)
$\sigma_{BCSFB}$	—	0.99	U(0,1)
$\sigma_{BBB}$	—	1	U(0,1)
$V_{P}$	l	3.12	U(0,100)
$k_{deg}$	1/h	26.60	U(0,100)
$V_{T_{E}}$	l	0.33	U(0,100)
$V_{T_{I}}$	l	11.09	U(0,100)
FR	—	0.71	U(0,100)
$L_{T}$	l/h	0.32	U(0.289,0.353)
$V_{T_{V}}$	l	1.68	U(0,100)

## 5. Results

We consider subsets of identifiable parameters for various threshold values η, based on the PSS results for the concentration in plasma $C_{P}$ in [8], to integrate PSS with the Bayesian inference. These subsets, their corresponding relative error norms at each threshold value and three criteria to determine whether Bayesian analysis is satisfactory are listed in table 2.

**Table 2.** PSS results for $C_{P}$ : $\bar{R}$ is the average relative error norm across the time domain of interest; (C1–C3) define the satisfactory performance of Bayesian inference (see §3 for details); ✓ (passed criteria); ✗ (failed to pass criteria).

η values	$\bar{R}$	parameter candidates $A=\left\{A_{1},A_{2},A_{3},A_{4},A_{5}\right\}$	C1	C2	C3
$2.5\times 10^{-8}$	0.15	$V_{P}$, $\sigma_{T_{V}}$, $k_{deg}$, $V_{T_{E}}$, $V_{T_{I}}$, FR	✗	✓	✗
$1\times 10^{-8}$	0.09	$V_{P}$, $\sigma_{T_{V}}$, $k_{deg}$, $V_{T_{E}}$, $V_{T_{I}}$, FR, $\sigma_{BCSFB}$	✗	✗	✗
$2.5\times 10^{-10}$	0.07	$V_{P}$, $\sigma_{T_{V}}$, $k_{deg}$, $V_{T_{E}}$, $V_{T_{I}}$, FR, $\sigma_{BCSFB}$, $\sigma_{BBB}$	✗	✗	✗
$1\times 10^{-10}$	0.10	$V_{P}$, $\sigma_{T_{V}}$, $k_{deg}$, $V_{T_{E}}$, $V_{T_{I}}$, FR, $\sigma_{BCSFB}$, $\sigma_{BBB}$, $L_{T}$	✗	✗	✗
$1\times 10^{-12}$	0.02	$V_{P}$, $\sigma_{T_{V}}$, $k_{deg}$, $V_{T_{E}}$, $V_{T_{I}}$, FR, $\sigma_{BCSFB}$, $\sigma_{BBB}$, $L_{T}$, $V_{T_{V}}$	✗	✗	✗

The results in [8] compare two PSS algorithms with quantitative and qualitative verification. To obtain subsets of parameter candidates, we employ an algorithm that recomputes the singular value decomposition by decreasing the number of columns in the sensitivity matrix at each iteration. Based on the result in [8], the higher relative error norm values are at the threshold value of $\eta =1\times 10^{-6}$ compared to other thresholds. Therefore, this threshold may not yield the most reliable identifiable parameter subset, and we skip Bayesian inference for this specific η. Also, any values smaller than $\eta =10^{-12}$ yield the same parameter candidates as $\eta =10^{-12}$. We have five sets of parameter candidates in total; therefore, we set k=5 in Algorithm 1. The DRAM algorithm is implemented in MATLAB using the MCMC toolbox, as outlined in [10,21]. The user must specify the number of iterations, an estimate of the measurement error, the covariance matrix, the prior distribution for each parameter and their initial values. Furthermore, since the system of ordinary differential equations for the mPBPK model is stiff, we use *ode15s* in MATLAB to solve it. We provide a refined subset of identifiable parameters, using the iterative method outlined in Algorithm 1, in table 3 for each threshold value. We detail the refinement of parameter subsets on three criteria in §5a–c.

**Table 3.** Refined subset of identifiable parameters obtained using Algorithm 1. After excluding parameters in the sixth column, all three criteria are satisfied.

η values	refined parameter candidates	C1	C2	C3	excluded parameter
$2.5\times 10^{-8}$	$V_{P}$, $\sigma_{T_{V}}$, $k_{deg}$, $V_{T_{E}}$, $V_{T_{I}}$, FR	✗	✓	✗	$V_{T_{E}}$
$1\times 10^{-8}$	$V_{P}$, $\sigma_{T_{V}}$, $k_{deg}$, $V_{T_{I}}$, FR, $\sigma_{BCSFB}$	✓	✗	✓	$\sigma_{BCSFB}$
$2.5\times 10^{-10}$	$V_{P}$, $\sigma_{T_{V}}$, $k_{deg}$, $V_{T_{I}}$, FR, $\sigma_{BBB}$	✓	✗	✓	$\sigma_{BBB}$
$1\times 10^{-10}$	$V_{P}$, $\sigma_{T_{V}}$, $k_{deg}$, $V_{T_{I}}$, FR, $L_{T}$	✓	✗	✓	$L_{T}$
$1\times 10^{-12}$	$V_{P}$, $\sigma_{T_{V}}$, $k_{deg}$, $V_{T_{I}}$, FR, $V_{T_{V}}$	✓	✓	✓	—

### (a) Threshold values of 2.5 × 10^−8^ and 1 × 10^−8^

**Figure 3.**
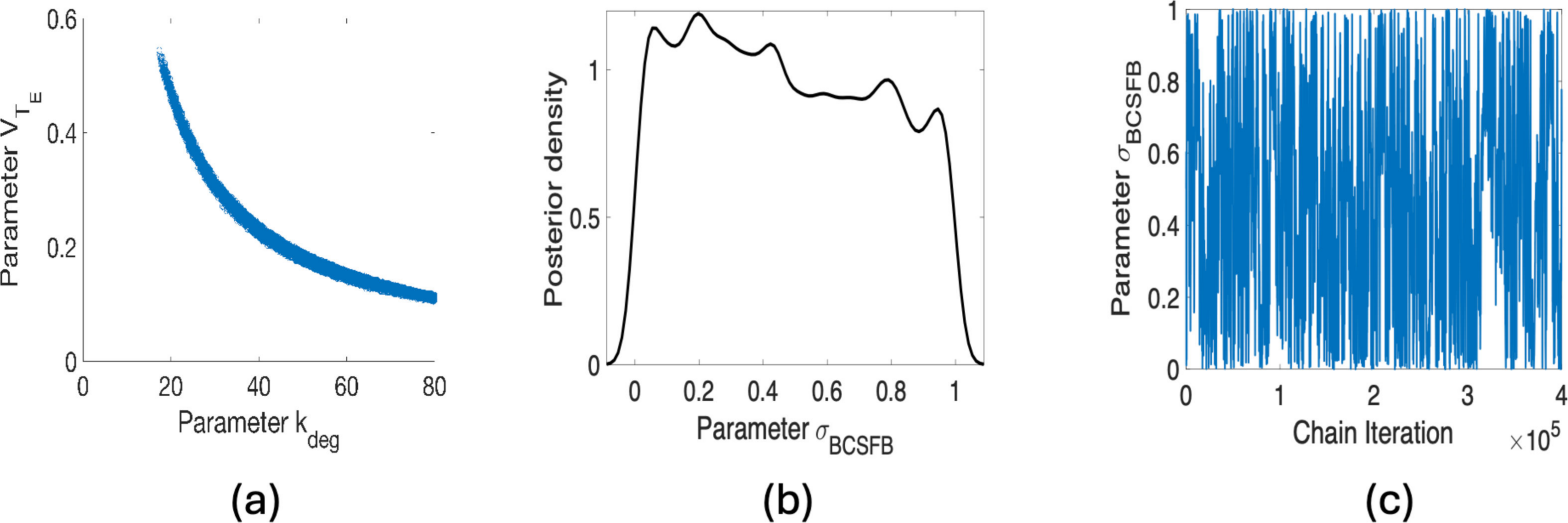
(*a*) Pairwise posterior distribution plot for the parameters $V_{T_{E}}$ and $k_{deg}$ for $\eta =2.5\times 10^{-8}$; (*b,c*) posterior distribution and chain plots for $\sigma_{BCSFB}$ for $\eta =10^{-8}$.

We consider first the subset $A_{1}$ of six parameters obtained using the threshold value of $\eta =2.5\times 10^{-8}$ as listed in table 2. When implementing Algorithm 1, we obtained a nearly single-valued plot between the parameters $V_{T_{E}}$ and $k_{deg}$ as shown in figure 3a. Whereas a general correlation between parameters does not prevent the convergence of MCMC methods, the single-valued posterior sample plot in figure 3a indicates that these two parameters are algebraically related and cannot be identifiable simultaneously; see criteria (C1) in §3.

We also list statistical diagnostics in table 4. The total number of simulations is denoted by M ($10^{6}$), and the term post-burn-in refers to ($4\times 10^{5}$), the total number of simulations after subtracting the burn-in period. In table 4, the mean is the average value of the parameter, and s.d. is a standard deviation of parameter estimates across MCMC iterations. The ESS is very low for all parameters, indicating poor chain mixing; recall that one seeks ESS values of 1000 or larger-see criteria (C3) in §3. Techniques to address this issue include the application of parallel chains, taking a large number of iterations, or employing more advanced MCMC sampling techniques.

**Table 4.** Posterior mean, posterior standard deviation and ESS of the MCMC chain for each parameter obtained with $\eta =2.5\times 10^{-8}$ .

parameter	mean	s.d.	ESS
$V_{P}$	3.12	0.06	130
$\sigma_{T_{V}}$	0.49	0.28	73
$k_{deg}$	64.85	40.73	10
$V_{T_{E}}$	0.20	0.11	34
$V_{T_{I}}$	16.45	2.95	91
FR	1.53	0.49	77

This case violates criteria (C1) in the iterative method described in §3. Based on the dimensional analysis presented in §3a, there is an inverse algebraic relation between $V_{T_{E}}$ and $k_{deg}$ from the ratio of two dimensionless quantities, i.e. the ratio of the first and the third quantities in equation (4.4), which is apparent in the shape of the posterior distribution in figure 3a.

**Figure 4.**
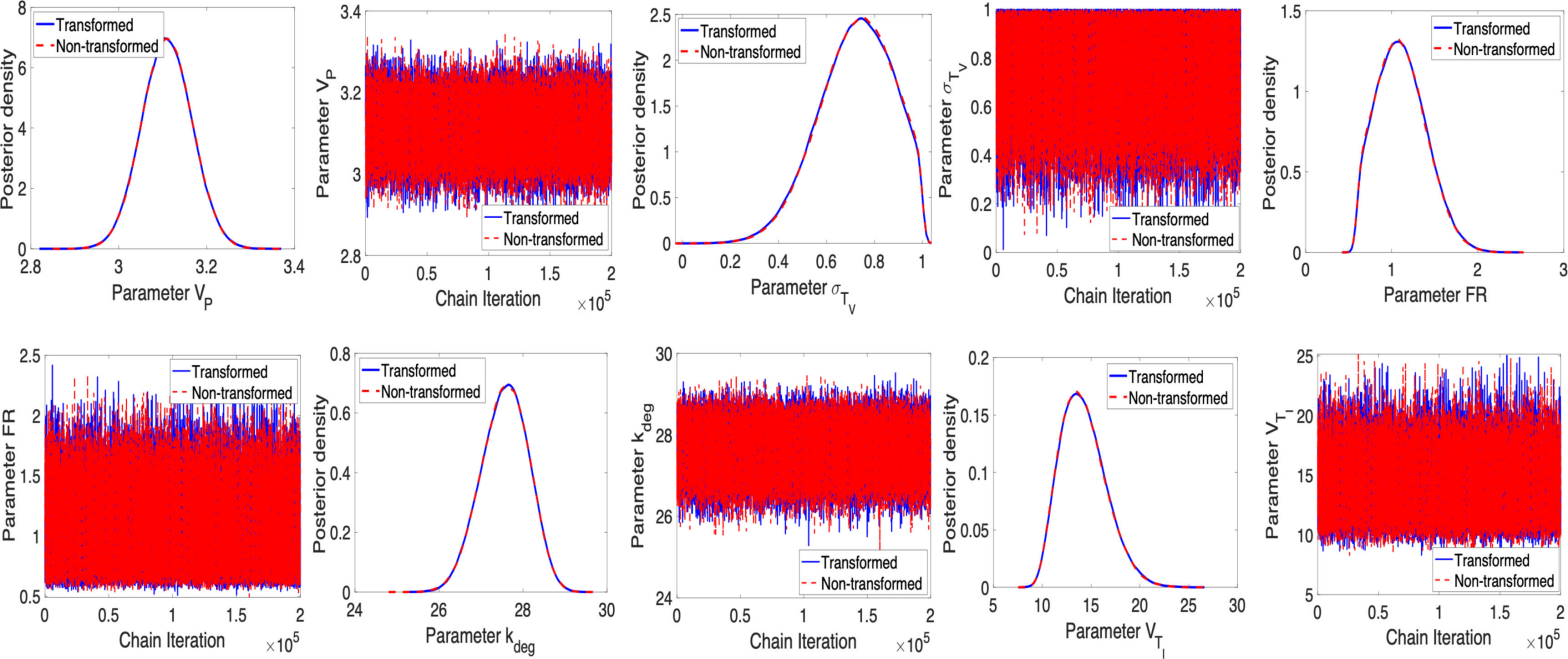
$\eta =2.5\times 10^{-8}$: Posterior density and parameter chains for parameters $V_{P}$, $\sigma_{T_{V}}$, FR, $k_{deg}$, $V_{T_{I}}$ for non-transformed and transformed cases obtained using unit conversion.

**Figure 5.**
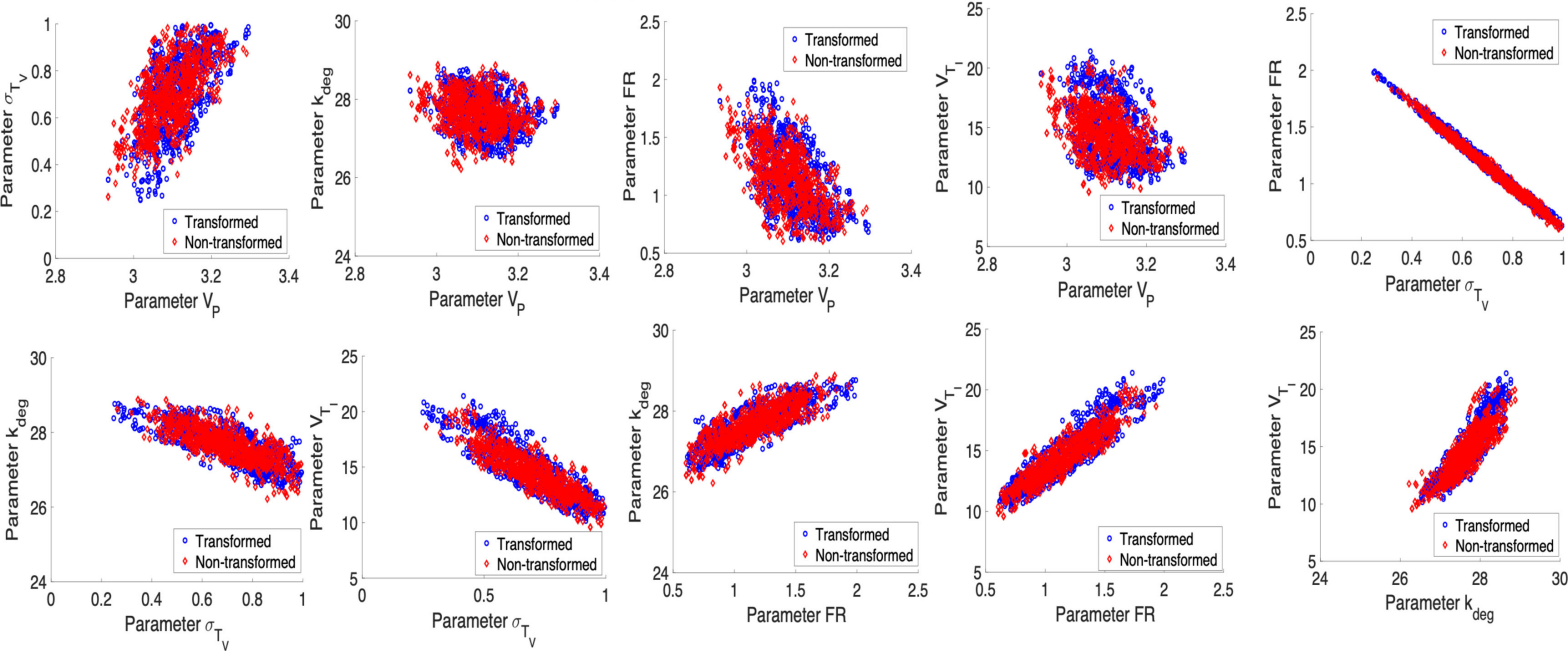
$\eta =2.5\times 10^{-8}$: Pairwise posterior plots for the parameters $V_{P}$, $\sigma_{T_{V}}$, FR, $k_{deg}$, $V_{T_{I}}$ for non-transformed and transformed cases obtained using unit conversion.

To address this issue, we follow the recommendation given in criteria (C1) by fixing $V_{T_{E}}$ at its nominal value. Note that we provide the case where $V_{T_{E}}$ is fixed at its nominal value and $k_{deg}$ is being treated as an estimated parameter. Furthermore, we have also checked the case when $k_{deg}$ is fixed at its nominal value and $V_{T_{E}}$ is estimated. Based on our observation, regardless of which parameter is fixed, both options solve the problem regarding sampling. The results are shown in figures 4 and 5. Furthermore, we assess parameter correlations primarily through visual inspection of pairwise posterior plots, but they can also be evaluated using the approximated covariance matrix.

We note that the posterior distributions for the parameters in figure 4 show that the model and data inform the prior distributions. Parameter chains in figure 4 are convergent, and we observe that the ESS values for defined parameters in table 5 are greater than 1000, indicating a good mixing and convergence for the parameters. The joint pairwise posterior distributions among the identifiable parameters are plotted in figure 5.

**Table 5.** Posterior mean, posterior standard deviation and ESS of the MCMC chain for each parameter obtained with $\eta =2.5\times 10^{-8}$ .

parameter	mean	s.d.	ESS
$V_{P}$	3.11	0.06	10 350
$\sigma_{T_{v}}$	0.72	0.15	8831
$k_{deg}$	27.57	0.55	8161
$V_{T_{I}}$	14.22	2.33	7550
FR	1.11	0.28	8576

**Figure 6.**
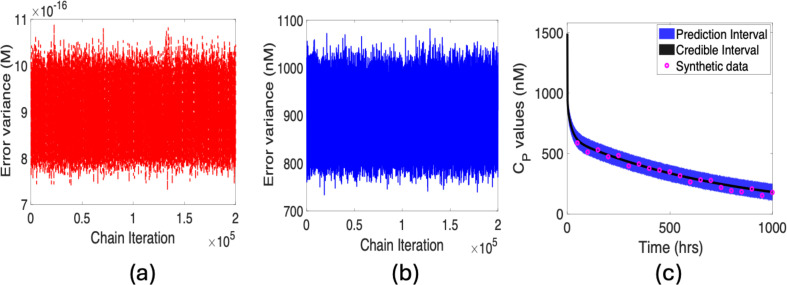
$\eta =2.5\times 10^{-8}$: Error variance plots for (*a*) non-transformed and (*b*) transformed cases; (*c*) prediction and credible intervals along with synthetic data.

A notable observation from figure 6a is that the magnitude of error variance is close to machine epsilon, which can yield potential numerical instability. We can address this by applying unit conversion as noted in §4; see figure 6b. This underscores the importance of scaling in the preliminary stage of Bayesian analysis to mitigate potential numerical issues. It also verifies the results without unit conversion.

In figure 6c, we plot the PPD for the concentration in plasma. We compute a credible interval that incorporates uncertainties owing to parameters. We also compute the 95% prediction interval, which incorporates parameter and observation uncertainties and quantifies the probability of future observations. To provide a clear visualization, we partition synthetic data into 50 distinct points that distinguish model predictions from the simulated data. As expected, the prediction interval is much wider than the credible interval. A narrow prediction interval indicates less uncertainty about the predicted values, implying that the model is robust about where future observations are likely to occur.

Based on these observations, all three criteria are now met for Bayesian inference for the subset of identifiable parameters corresponding to $\eta =2.5\times 10^{-8}$, and we can analyse the next subset in table 3. For the threshold value of $\eta =1\times 10^{-8}$, the parameter $\sigma_{BCSFB}$ is added to the subset of candidate parameters (see $A_{2}$). Based on the outcome in figure 3b,c, the data and model do not inform the prior distribution of this parameter, which leads to failing to pass criteria (C2) in the iterative method. Therefore, we remove parameter $\sigma_{BCSFB}$ from a subset of parameters and fix it at its nominal value.

### (b) Threshold values of 2.5 × 10^−10^ and 1 × 10^−10^

**Figure 7.**
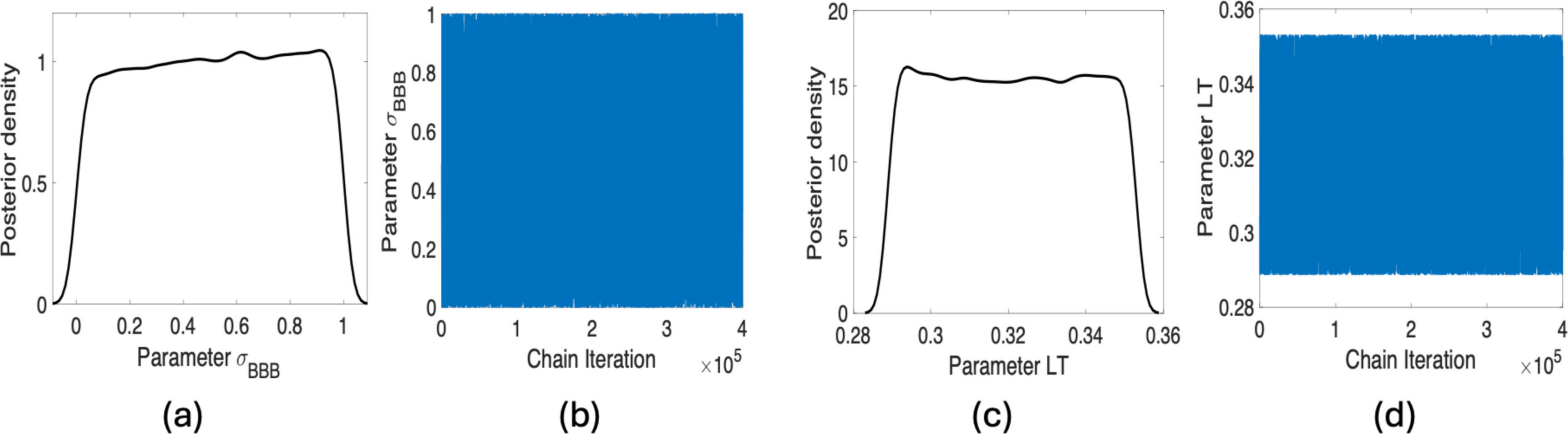
(*a–c*) Posterior distributions; (*b–d*) chain plots for the parameters for $\sigma_{BBB}$ and $L_{T}.$

We subsequently focus on the subset of parameter candidates $A_{3}$ for $\eta =2.5\times 10^{-10}$ for the concentration in plasma. This case introduces the parameter $\sigma_{BBB}$, yielding six parameters. Based on the results in figure 7*a*,b, the model and data do not inform the prior distribution of $\sigma_{BBB}$; therefore, this parameter is not identifiable, and we fix it at its nominal value by removing it from the subset of parameters to satisfy criteria (C2) in the iterative method.

Following this analysis, we analyse the subset corresponding to a threshold value of $\eta =1\times 10^{-10}$, which includes the parameter $L_{T}$ (see $A_{4}$ in table 2). Note that we initially chose the prior distribution as a uniform distribution with bounds of 0 and 100; sampled values yielded non-physical $L_{T}$ values. This issue could be addressed using a tighter prior, such as uniform distribution within an interval of 10% perturbations about the nominal value of $L_{T}$, defined in table 1. For the tighter prior distribution for $L_{T}$, we obtain the chains and marginal distributions plotted in figure 7c,d. Based on these plots, the model and data do not inform the prior distribution of $L_{T}$; hence, this parameter is not identifiable and is fixed at its nominal value.

Since these modifications yield the same identifiable parameter subset obtained in §5a, the results are identical to those obtained for the threshold value of $2.5\times 10^{-8}$, which are plotted in figures 4–6. Therefore, we do not provide plots for this case.

### (c) Threshold value of 1 × 10^−12^

**Figure 8.**
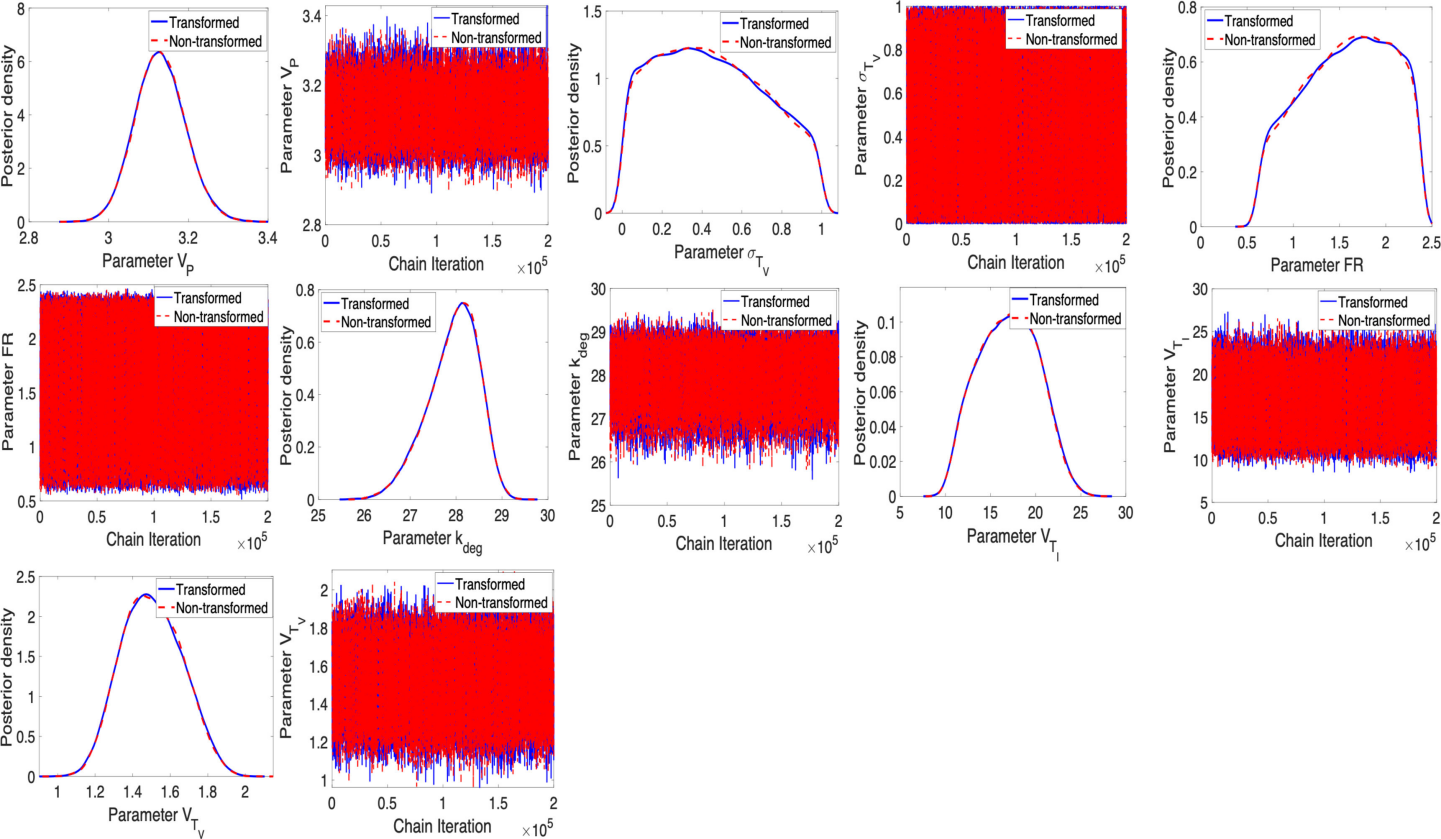
$\eta =10^{-12}$: Posterior density and parameter chains for parameters $V_{P}$, $\sigma_{T_{V}}$, FR, $k_{deg}$, $V_{T_{I}}$, $V_{T_{V}}$ for non-transformed and transformed cases.

**Figure 9.**
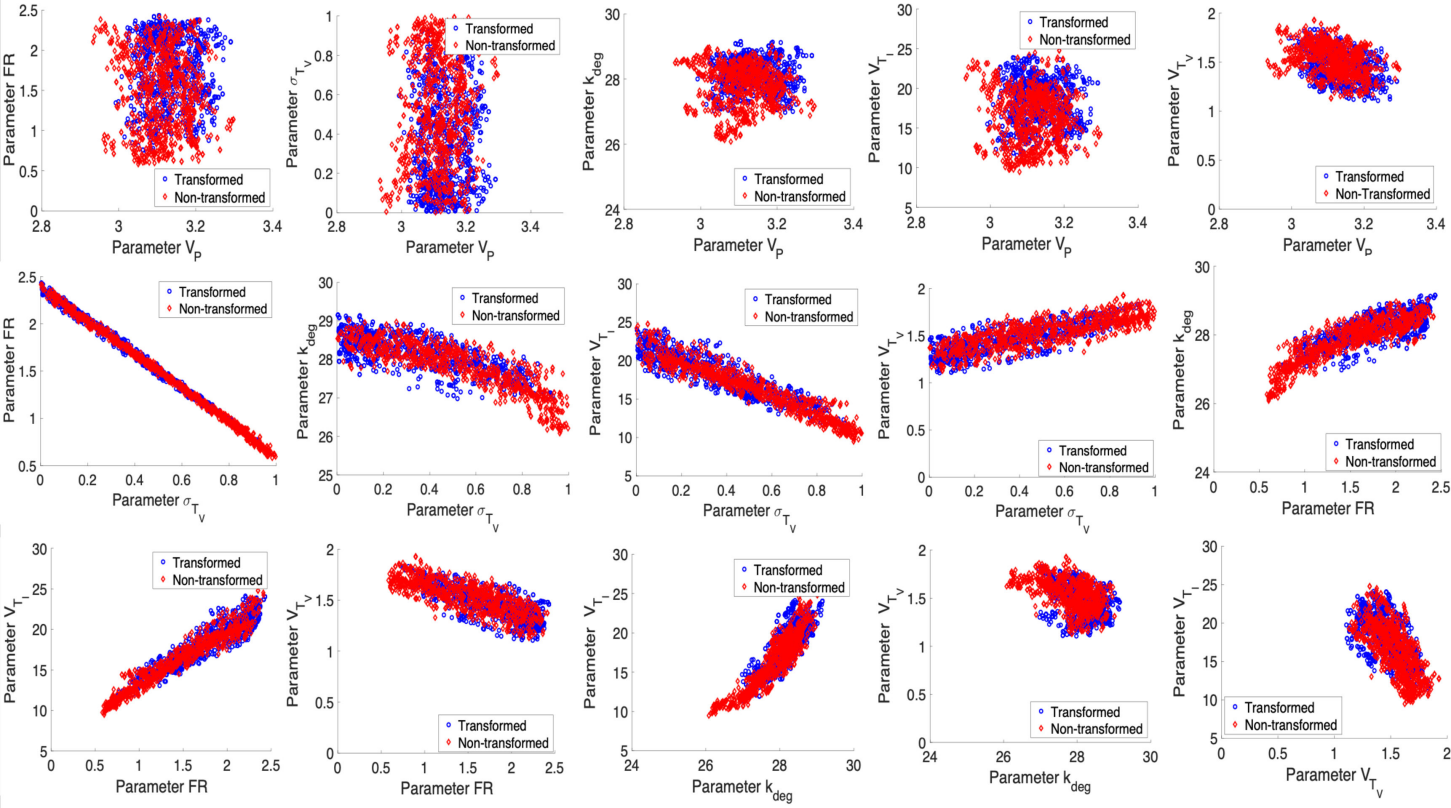
$\eta =10^{-12}$: Pairwise posterior plots for the parameters $V_{P}$, $\sigma_{T_{V}}$, FR, $k_{deg}$, $V_{T_{I}}$, $V_{T_{V}}$ for non-transformed and transformed cases.

**Figure 10.**
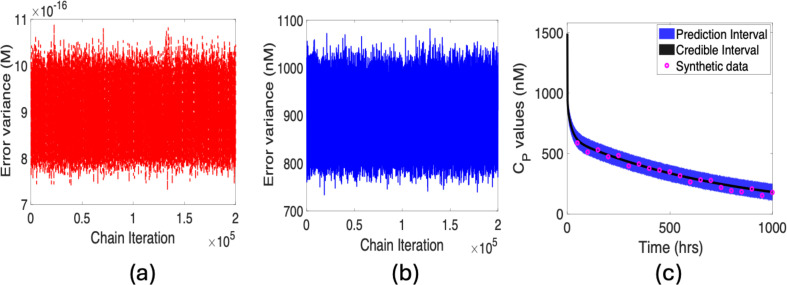
$\eta =10^{-12}$: Error variance plots for (*a*) non-transformed and (*b*) transformed cases; (*c*) prediction and credible intervals along with synthetic data.

Finally, we consider the subset of parameters for the threshold value of $\eta =10^{-12}$. This introduces one new parameter $V_{T_{V}}$ (see $A_{5}$ in table 2), making a final subset of $V_{P}$, $\sigma_{T_{V}}$, FR, $V_{T_{I}}$, $k_{deg}$ and $V_{T_{V}}$. Results are provided in figures 8–10.

Based on the results for marginal distributions and trace plots of the parameters in figure 8, the posterior distributions of the parameters are informed by the model and data. The chains for the parameters for this prescribed threshold value are convergent. Plotted joint distributions in figure 9 do not reveal single-valued behaviour among parameters. Based on these results, we conclude that the subset of identifiable parameters defined for this threshold value passes all three criteria in our iterative method. The associated plots for the error variance, prediction, and credible intervals for the response $C_{P}$, along with synthetic data, are provided in figure 10.

## 6. Discussion and conclusions

In this investigation, we integrate PSS, and use dimensional analysis and Bayesian inference to yield a subset of parameters that can be employed for subsequent uncertainty quantification. We achieve this through an iterative refinement process in which we employ PSS to provide initial subsets of parameters that we incrementally increase based on criteria that reflect successful Bayesian inference. This is complemented by dimensional analysis to determine potential algebraic dependencies among parameters.

Using this algorithm, we reduced the initial subset of 10 parameters to a final subset of six identifiable parameters for which we can compute distributions and construct prediction intervals for the considered responses. We achieve this by determining and fixing parameters that demonstrate algebraic dependencies as exhibited by nearly single-valued joint distributions which we further verify via dimensional analysis. We also determined that certain switch parameters were non-identifiable in the sense that they yielded model responses that did not inform prior distributions. We subsequently fix these parameters at their nominal values. This is consistent with their role in the model as dimensionless parameters that determine the partitioning of concentrations between adjacent compartments. This provides a potential framework for subsequent model reduction. Finally, we emphasize the importance of employing units, which provide scales that are robust for identifiability analysis, Bayesian inference and uncertainty propagation.

The success of the method presented in this paper depends on the accuracy of the initial subsets of parameters based on PSS [8]. In the case of a poorly constructed initial parameter subset, our iterative procedure could yield an empty set, indicating that no subset is consistent with successful Bayesian inference. In this case, the PSS procedure should be revisited prior to applying this approach. Our proposed algorithm is applicable to any mechanistic model that efficiently supports PSS and Bayesian inference. In addition, changes in the time domain of interest will affect the outcomes of PSS, thereby altering the refined parameter candidates within the Bayesian framework.

As with all Bayesian methods, the feasibility of sampling methods can diminish as parameter dimensions increase. Whereas this method reduces the number of considered parameters, additional analysis may be required for models having moderate to high parameter dimensions. One method under current investigation is Bayesian model averaging, a technique that combines predictions from multiple models by averaging them based on their probabilities, which can potentially improve efficiency when accounting for model uncertainty [13]. This method screens with the PSS algorithm to determine the largest subset of parameter candidates and then employs probabilistic analysis to determine a feasible parameter subset.

## Data Availability

Data are available on Zenodo [22].
